# *Duox*, *Flotillin-2*, and *Src42A* Are Required to Activate or Delimit the Spread of the Transcriptional Response to Epidermal Wounds in *Drosophila*

**DOI:** 10.1371/journal.pgen.1002424

**Published:** 2011-12-29

**Authors:** Michelle T. Juarez, Rachel A. Patterson, Efren Sandoval-Guillen, William McGinnis

**Affiliations:** Section of Cell and Developmental Biology, Division of Biology, University of California San Diego, La Jolla, California, United States of America; Stanford University School of Medicine, United States of America

## Abstract

The epidermis is the largest organ of the body for most animals, and the first line of defense against invading pathogens. A breach in the epidermal cell layer triggers a variety of localized responses that in favorable circumstances result in the repair of the wound. Many cellular and genetic responses must be limited to epidermal cells that are close to wounds, but how this is regulated is still poorly understood. The order and hierarchy of epidermal wound signaling factors are also still obscure. The *Drosophila* embryonic epidermis provides an excellent system to study genes that regulate wound healing processes. We have developed a variety of fluorescent reporters that provide a visible readout of wound-dependent transcriptional activation near epidermal wound sites. A large screen for mutants that alter the activity of these wound reporters has identified seven new genes required to activate or delimit wound-induced transcriptional responses to a narrow zone of cells surrounding wound sites. Among the genes required to delimit the spread of wound responses are *Drosophila Flotillin-2* and *Src42A*, both of which are transcriptionally activated around wound sites. *Flotillin-2* and constitutively active *Src42A* are also sufficient, when overexpressed at high levels, to inhibit wound-induced transcription in epidermal cells. One gene required to activate epidermal wound reporters encodes *Dual oxidase*, an enzyme that produces hydrogen peroxide. We also find that four biochemical treatments (a serine protease, a Src kinase inhibitor, methyl-ß-cyclodextrin, and hydrogen peroxide) are sufficient to globally activate epidermal wound response genes in *Drosophila* embryos. We explore the epistatic relationships among the factors that induce or delimit the spread of epidermal wound signals. Our results define new genetic functions that interact to instruct only a limited number of cells around puncture wounds to mount a transcriptional response, mediating local repair and regeneration.

## Author Summary

An epidermal wound provides signals that initiate a variety of localized responses, some of which act to regenerate and repair the breach in the epidermal barrier. The *Drosophila melanogaster* embryonic epidermis provides an excellent system to discover new genes that regulate wound-healing processes. Using fluorescent epidermal “wound” reporters that are locally activated around wound sites, we have screened almost 5,000 *Drosophila* mutants for functions required to activate or delimit wound-induced transcriptional responses to a local zone of epidermal cells. Among the seven new genes required to delimit the spread of wound responses are *Flotillin-2* and *Src42A*. These two genes are also sufficient, when overexpressed at high levels, to inhibit wound-induced transcription in epidermal cells. One new gene required to activate epidermal wound reporters encodes *Dual oxidase*, an enzyme that produces hydrogen peroxide. We also find that four biochemical treatments (a serine protease, a Src kinase inhibitor, methyl-ß-cyclodextrin, and hydrogen peroxide) are sufficient to globally activate epidermal wound response genes in *Drosophila* embryos. Our results define new genetic functions, and the interactions among them, which regulate the local transcriptional response to puncture wounds.

## Introduction

The development of a specialized epidermal barrier layer represents a key step during the evolution of multi-cellular organisms. This outer integument provides protection from the environment and helps maintain cellular homeostasis. Epidermal barriers consist of epithelial cells that are tightly joined by adherens and other types of junctional complexes, as well an apical extracellular matrix layer that is highly variable. The mammalian epidermal barrier is constructed from a constantly renewing multicellular layer, in which cells follow a complex process of cell division and differentiation to form the stratum corneum [1]. In arthropods like *Drosophila melanogaster*, a single epidermal cell layer secretes a multilayered matrix of cross-linked lipid, protein, and chitin to generate a largely impermeable cuticle barrier [2], [3]. Despite the great differences between the components and physical makeup of their epidermal barriers, both mammals and arthropods make use of conserved cellular mechanisms, transcriptional regulators, and signaling pathways during the generation of epidermal barriers as well as during their regeneration after wounding [4], [5], [6], [7]. There are many complex processes that contribute to epidermal wound healing; these include clot formation, reepithelialization, cellular proliferation, inflammation, and barrier replacement [8]. *Drosophila* is a genetically tractable system for discovering evolutionarily conserved genes involved in such epidermal wound healing processes, as it has been for discovering genes that regulate animal septic wound responses [9].

One useful system for elucidating cellular mechanisms involved in wound healing has been *Drosophila* dorsal closure—where sheets of embryonic epidermal cells migrate to join at the dorsal midline—which uses some of the same cellular processes that are used to heal wounds [10], [11]. For example, both dorsal closure and wound healing involve the recruitment of an actin-cytoskeleton “purse-string” to help close the edge of the wound or the edge of a gap in a migrating dorsal epidermal sheet [12]. Several evolutionarily conserved transcriptional regulatory pathways have been linked to developmental control of barrier formation as well as wound healing [13]. For example, Grainy head (Grh) transcription factors are required in a variety of animals for the development of impermeable epidermal barriers as well as normal wound repair [5], [6], [14], [15], [16], [17]. In *Drosophila*, Grh accomplishes these functions in part through regulation of the *Dopa Decarboxylase (Ddc)* and *Tyrosine hydroxylase (ple)* genes, which encode enzymes that produce cuticle protein cross-linkers [5], [18].

Other transcription factors with conserved roles in wound repair are those in the JUN family, which are required for wound reepithelialization in both mammals and *Drosophila* [7], [19]. Upstream of JUN, the JUN amino-terminal kinase (JNK) signaling pathway [20] is required in the *Drosophila* epidermis for dorsal closure and wound reepithelialization [4], [21], [22]. *misshapen* (*msn*), which encodes a *Drosophila* JNK-kinase-kinase-kinase, is distinctive because it is transcriptionally activated around embryonic, larval and adult epidermal wounds [4], [21], [22], [23], [24], [25]. Recent reports have shown that JNK signaling is also required during *Drosophila* wing imaginal disc regeneration [26], [27]. Another signaling pathway involving the gene *stitcher* (*stit*), which encodes a receptor tyrosine kinase, is also activated around epidermal wound sites in *Drosophila* embryos, and is required for normal wound reepithelialization, and activation of some epidermal barrier repair genes [28].

There have been a few focused genetic screens for *Drosophila* mutants required for normal epidermal wound repair. One was a screen of 665 P-element insertional mutants for abnormal wound reepithelialization phenotypes after laser wounding during embryogenesis. The mutations with the most severe defects were in the genes for the JUN transcription factor and ß_Heavy_ Spectrin [7]. Interestingly, wound closure defects were not observed in several genes that are required for dorsal closure and epithelial migration during *Drosophila* development, indicating that wound closure and dorsal closure are, to some extent, under the control of distinct genetic systems [7]. Another *Drosophila* genetic screen used a combination of dominant negative and RNAi-mediated knockdowns to test about 180 genes, focusing on Receptor Tyrosine Kinases (RTKs), JNK signaling components, and cytoskeletal components after pinch or puncture wounding of the larval epidermis [22], [29]. The knockdown or knockout of function in about 20 genes showed defective reepithelialization after larval wounding. Genes required for normal larval reepithelialization include those encoding components of the JNK signaling pathway like the transcription factors JUN (*Drosophila Jra*) and FOS (*Drosophila kay*), as well as the *Drosophila* PDGF/VEGF-like receptor (*Pvr*), and some proteins that regulate or remodel the actin cytoskeleton [22], [29]. A few of the tested genes (encoding JUN, JNK, and JUNKK, respectively) were also required for the transcriptional activation of a wound response reporter gene (*misshapen-lacZ*) in larval epidermal cells surrounding sterile wound sites [22].

We have initiated a large, unbiased, genetic screen to identify mutations that are required for localized activation of epidermal genes around clean puncture wounds in *Drosophila* embryos. At this point, there are cis-regulatory wound enhancers identified from the *Ddc*, *ple*, *msn*, *kkv*, and *stit* genes [25], [28]. These enhancers, when attached to fluorescent reporter genes (hereafter called wound reporters) provide a visible readout of wound-induced gene activation after epidermal wounding, and can be used to identify mutations that are required to activate or localize (delimit) this response. A few hours after wounding late stage embryos, fluorescent signal from these epidermal wound reporters can be observed in a zone that extends ∼5–10 cells from puncture sites. Some particularly interesting regulatory genes that we discuss in this paper are those required to delimit or localize the activity of wound reporters to a zone within a few cell diameters from wound sites. Mutations in such genes result in a global activation of wound reporters in most or all epidermal cells after wounding. One of the wound localization genes we identified is *reggie-1/Flotillin-2* (referred to as *flotillin-2* or *Flo-2* in Flybase), which was originally isolated as a gene that is activated in wounded, regenerating goldfish neurons [30], and as a protein enriched in lipid rafts [31]. At the cellular level, Flo-2 appears to be involved in a variety of cell signaling and adhesion functions, at least in part via its role in clathrin-independent vesicular trafficking [32], [33], [34], [35], [36]. Analogous to wounded fish neurons, the *Flo2* gene in *Drosophila* embryos is transcriptionally activated in cells surrounding epidermal wounds. We find that *Flo-2* interacts in a pathway involving *Drosophila Src42A* to delimit epidermal wound responses, and that overexpression of either *Flo-2* or activated *Src42A* can inhibit wound reporter activation, whether it is triggered locally by epidermal puncture, or globally by injection of trypsin or hydrogen peroxide.

## Results

**Figure 1:**
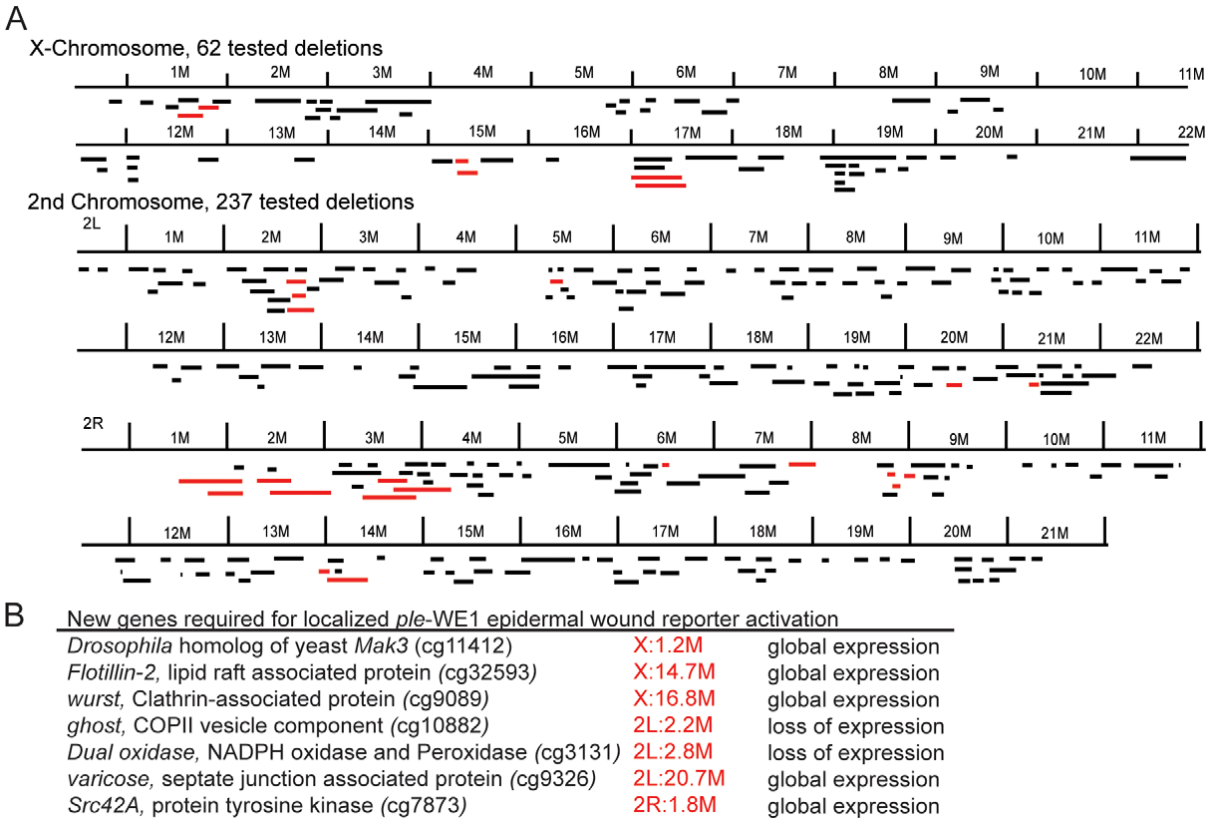
Summary of epidermal wound reporter mutants and their phenotypes. (A) Maps of X and 2nd chromosomes. Black bars represent deleted regions that have a normal epidermal wound reporter phenotype. Red bars represent deleted regions that have an altered epidermal wound reporter express pattern. (B) A list of new genes identified that affect the localization of the epidermal wound reporter. X:1.2M, for example, denotes 1.2 Megabases on the *Drosophila* genome map of the X chromosome [93].

Monitoring the activity of epidermal wound reporters in late stage *Drosophila* embryos [25] provides an *in vivo* assay that can be used to screen for mutations that are required to activate or localize the expression of genes that respond to epidermal wounding. We began such a screen using a collection of well-defined small chromosomal deletions of the *Drosophila melanogaster* genome [37], searching for regions containing zygotic functions required for normal activation of the wound reporter *ple*-WE1 [25]. One advantage of this screen is that most *Drosophila* zygotic mutants survive to late stages of embryogenesis, differentiate their epidermis (which can be assayed by the activation of an anal pad specific enhancer that exists alongside the wound enhancer within the *ple*-WE1 sequence), and can still be assayed for wound reporter activity. At this point, we have screened 300 deletions that include approximately 4,600 genes on the X and 2nd chromosomes (Figure 1A). Sixteen of these deletions had abnormal *ple*-WE1 expression, and therefore contained putative epidermal wound regulatory genes. Analysis of the genes within the collection of deletions indicates that the zygotic functions of many signaling pathways, transcription factors, and other regulators of cellular properties have no effect on the activation of the *ple*-WE1 epidermal wound reporter (Table 1). For example, deletion mutants of *patched* (Hedgehog pathway), *shaggy*/GSK3 or *disheveled* (Wingless pathway), Notch (Notch signaling pathway), *Pvr* (PDGF/VEGF signaling pathway), *domeless* (JAK/STAT pathway), and *wengen* (TNF pathway) all showed normal *ple*-WE1 expression after wounding. It is possible that the maternal contribution of some of these genes is sufficient to rescue potential effects on wound reporter activation in zygotic mutants.

**Table 1:** Zygotic mutants in these genes show *ple*-WE1 wound reporter expression in a normal zone in the epidermis around wounds.

Gene name	Signaling pathway or process eliminated
*Dof*	FGF receptor pathways
*Pvr*	Drosophila PDGF/VEGF signaling pathway
*Pvf1*	Ligand for Pvr
*Jra/JUN*	JUN transcription factor
*bsk/JNK*	JUN Kinase pathway
*fused, smoothened*	Hedgehog signaling pathway
*patched*	Hedgehog receptor
*Toll, imd, Dif, dl*	Innate immune pathways
*wengen*	Tumor necrosis factor/eiger receptor
*hopscotch, domeless*	JAK/STAT signaling pathway
*shg/GSK-3*	Wg/Wnt canonical signaling pathway
*Wnt10, Wnt2*	Wg/Wnt ligand
*dishevelled*	Wingless signal transduction, canonical and non-canonical
*armadillo/ß-catenin*	Cadherin protein complex, canonical Wnt signaling pathway
*shark*	One branch of Src-family signaling pathways in *Drosophila*
*Btk29A*	One branch of Src-family signaling pathways in *Drosophila*
*minibrain/Dyrk*	One branch of Src-family signaling pathways in *Drosophila*
*Notch*	Notch signaling pathway
*Flotillin-1*	Membrane associated protein, component of lipid raft
*starry night*	GPCR, establishment of planar cell polarity
*pickle/megatrachea*	Claudin-like, component of septate junction
*Sec24A/B*	COP II vesicle trafficking
*hippo, yorkie*	Cell proliferation
*Dsor*	MAPKK in RTK signaling pathways
*IP3K1*	Regulation of Ca^++^ homeostasis
*rab5*	GTPase, regulation of endocytosis
*moesin*	ERM protein family, actin cytoskeleton

Within the 16 regions required for normal epidermal wound reporter expression, we have currently defined 7 single gene mutations with new functions in wound reporter activation or localization (Figure 1B). The functions of two of these genes (*ghost/stenosis, and Duox*) are required for wound gene activation, and five of the genes (*flotillin-2*, *wurst*, *varicose*, *Src42A*, and the *Drosophila* homolog of yeast *MAK3*) are required to localize wound reporter activity to the immediate vicinity of wounds. One focus of this paper is on *flotillin-2 (Flo-2)*. The Flo-2 protein has been well characterized at the cellular and biochemical levels, but the genetic interactions of *Flo-2* are still enigmatic in the diverse cellular processes in which it participates [38]. The *Drosophila* genome encodes only one Flo-2 ortholog [39]. Null mutants that eliminate *Drosophila* Flo-2 protein also accumulate little or none of the related Flo-1 protein, since it is apparently destabilized in the absence of Flo-2 [40]. *Flo-2* mutant animals have normal morphology and are viable and fertile [40]. Despite having normal adult morphology, *Flo-2* mutants show a reduced spread of Wnt and Hedgehog signals in wing imaginal discs [40], [41]. *Flo-1* mouse mutants are viable and fertile under standard lab conditions and the mutants have somewhat reduced Flo-2 protein levels [42]. We tested a *Drosophila* deletion mutant that eliminated the *Flo-1* gene, but *ple*-WE1 expression was normal after wounding (Table 1).

**Figure 2:**
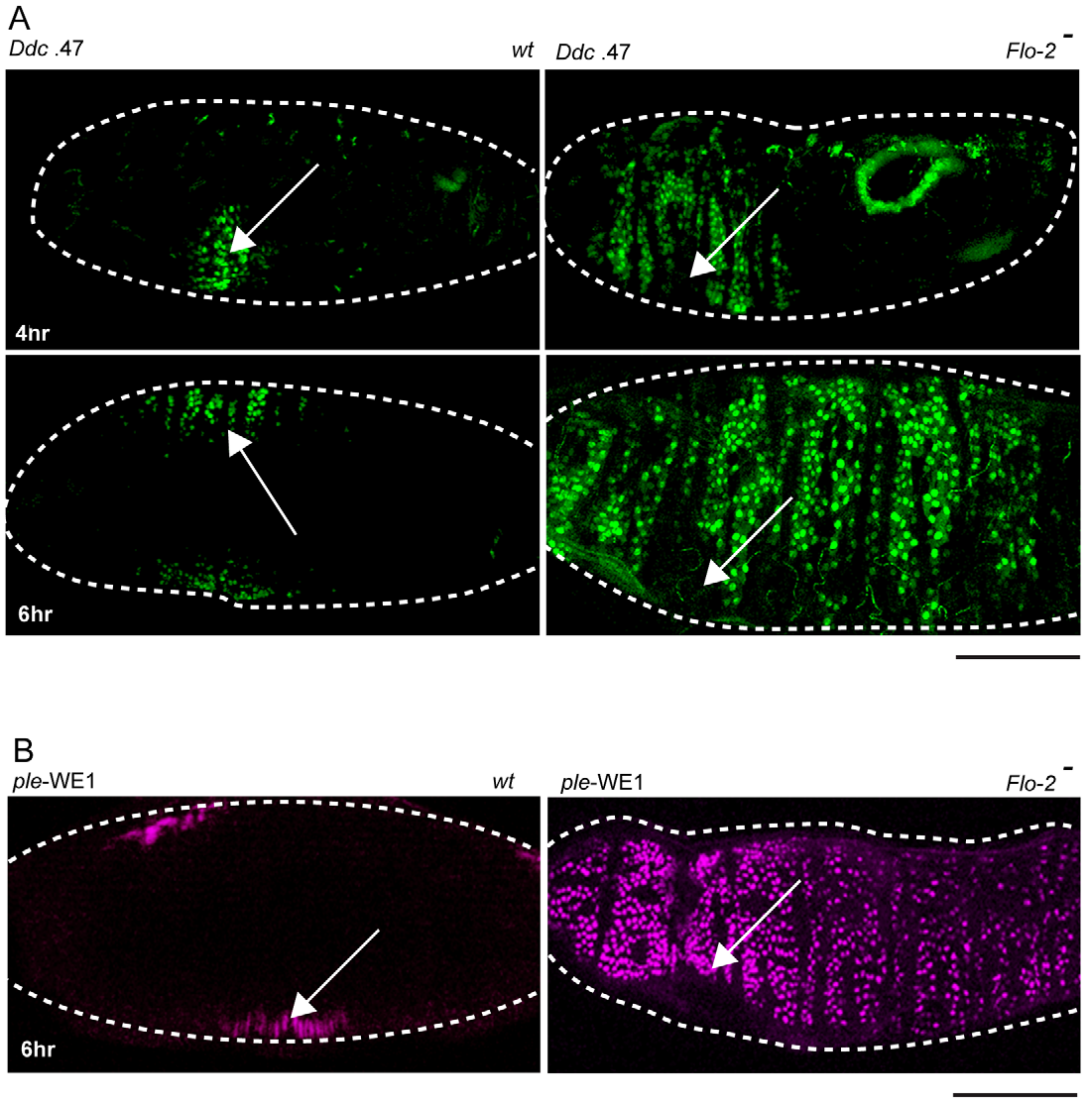
*Flo-2* functions to inhibit widespread activation of epidermal wound reporters. (A) Confocal images of *Ddc* .47-GFP epidermal wound reporter activity (4–6 hours after puncture wounding). Wild type (*wt*) embryos show the reporter in cells around the wound site. *Flo-2* mutant embryos show expansion of *Ddc* .47 reporter expression to most epidermal cells. (B) *ple*-WE1-dsRed epidermal wound reporter expression expands to most epidermal cells in *Flo-2* mutant embryos 6 hours after wounding. Arrows mark the site of the wound. Dashed lines in the data panels mark the outlines of embryos. Scale bars = 50 µM.

Normal epidermal activation of our fluorescent protein wound reporters can be easily detected 4 hours after wounding wild type stage 16 embryos (Figure 2A, 2B), although transcripts from the same endogenous wound-activated genes in a narrower zone of cells can be detected in fixed embryos within 30 minutes after wounding [5], [25]. The fluorescent protein reporters have the great advantage of being easily detectable in living embryos or larvae, whereas nucleic acid or antiserum probe permeability into late stage embryos or larvae with partially or fully differentiated cuticle is labor intensive at best. However, the fluorescent protein reporters typically represent a delayed version (by our estimate, a few hour delay) of the transcriptional response in cells surrounding wounds. This is due to the requirement of enhanced-GFP or enhanced-dsRed proteins to oxidatively mature to fluorescence. We estimate that the fluorescent reporter proteins we used [5], [25], [43]. have a half-time to maturation of about an hour in fly embryos after the reporter gene RNAs are translated. Additionally, time is required to accumulate detectable levels of fluorescent protein, and this is dependent on the strength of the epidermal wound enhancer being tested. For example, the *Ddc* .47 wound enhancer appears to be slightly stronger than the *ple*-WE1 enhancer [5], [25].

In *Flo-2* mutants, fluorescent wound reporter proteins driven by the *ple*-WE1 and *Ddc* .47 wound enhancers are detected at 4 hours after wounding, but reporter expression is significantly expanded compared to wild type embryos (Figure 2A, 2B). By 6 hours post-wounding reporter expression in *Flo-2* mutants spreads to include most epidermal cells in late embryos (Figure 2A, 2B). Using *in situ* hybridization or protein immunodetection, we also tested the effect of *Flo-2* mutants on the activation pattern of several endogenous wound response genes (*ple*, *Ddc*, *msn*, *stit*, and *Src42A*), and found comparable expanded expression domains after wound induction (Figure S1, and data not shown). Nearly all (∼90%) of wounded *Flo-2* mutant embryos survive, hatch to become larvae, and progress to adulthood, which was similar to that observed in wounded wild type embryos (data not shown). We did not detect any characteristics of abnormal epidermal wound healing [26], or ectopic melanization in the punctured *Flo-2* mutants, although we did not carefully examine the kinetics of healing in the mutants. Activation of wound reporters in *Flo-2* mutants was wound-dependent; in no instances was constitutive expression of reporters detected in mutant embryos.

**Figure 3:**
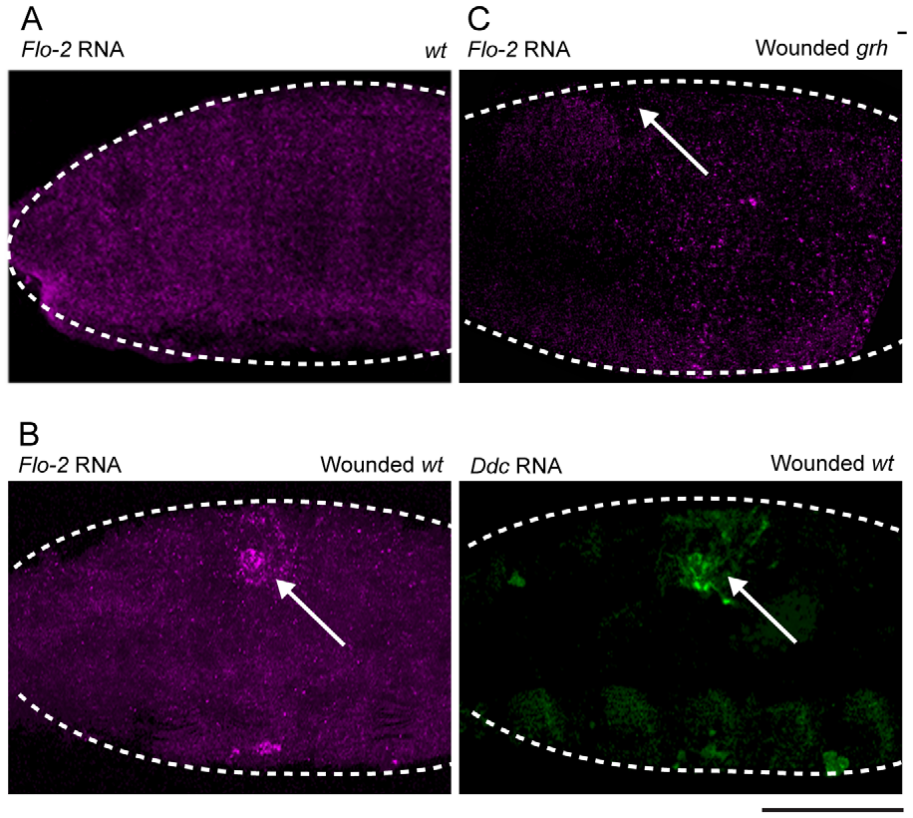
*Flo-2* transcriptional activation at wound site depends on *grainy head* function. Confocal images of *in situ* hybridizations with fluorescently labeled probes made from *Flo-2* and *Ddc* cDNA clones. (A) *Flo-2* transcripts accumulate in all epidermal cells of late stage embryos. (B) 30-minutes after puncture wounding, *Flo-2* transcripts show increased accumulation in cells around wound sites, in a similar pattern as wound-induced *Ddc* transcript activation in the epidermis. (C) 30-minutes after puncture wounding, *grainy head* (*grh*) mutant embryos fail to show increased accumulation of *Flo-2* transcripts around epidermal wound sites. Arrows mark the wound site. Dashed lines in the data panels mark the outlines of embryos. Scale bar = 50 µM.

*Flo-2* transcripts normally accumulate in all cells of the embryo, including the epidermis (Figure 3A), with higher apparent levels in the central nervous system [40], [41]. The insertional mutant into *Flo-2 (Flo-2[KG00210])* used in this study fails to accumulate transcripts (Figure S1A; [40]). A 500 bp region within the largest of the *Flo-2* introns contains predicted high affinity binding sites for the Grh, AP-1, and ETS transcription factors. Such sites are found in clusters in previously characterized epidermal wound response enhancers from the *ple*, *Ddc*, msn, and *stit* genes [25], [28]. To test whether *Flo-2* is transcriptionally activated around epidermal wounds, we carried out *in situ* hybridizations on wounded embryos using a *Flo-2* probe. As seen in Figure 3B, *Flo-2* transcripts are expressed at higher levels in cells surrounding epidermal wound sites, overlapping with the wound induced activation of the endogenous *Ddc* gene. Previous studies showed the activation of some epidermal wound response genes depends on the function of the Grh transcription factor [25], [28]. As seen in Figure 3C, *Flo-2* transcriptional activation around epidermal wound sites is dramatically reduced in *grh* mutant embryos compared to wild type siblings (Figure 3B), consistent with *Flo-2* being activated in a Grh-dependent manner.

**Figure 4:**
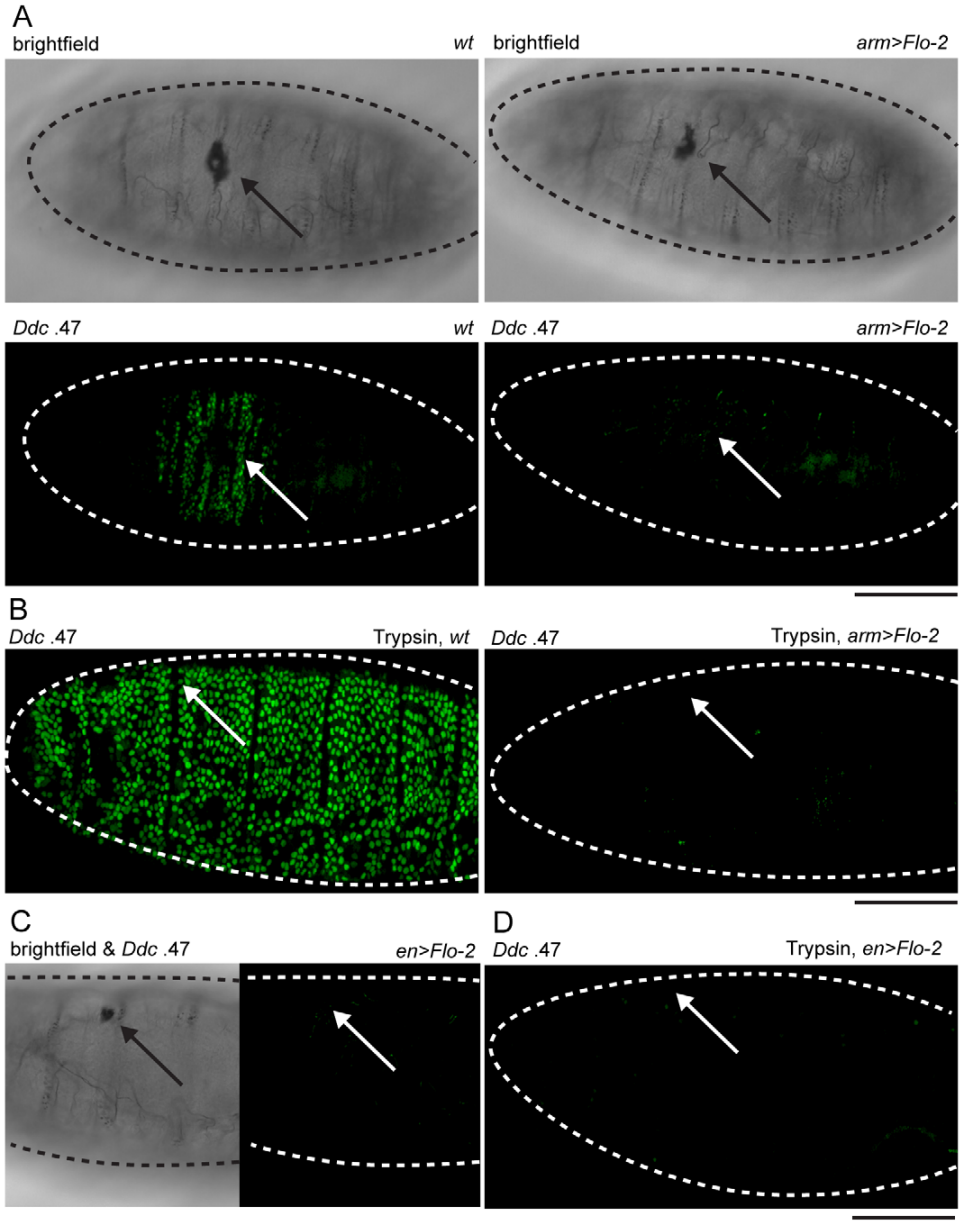
Overexpression of *Flo-2* inhibits activation of an epidermal wound reporter. (A) Brightfield and fluorescent confocal images of late stage embryos with *Ddc* .47-GFP epidermal wound reporter genes. Wild type (*wt*) embryos, or embryos with overexpressed *Flo-2* (*arm>Flo-2*) have similar melanized clots in the cuticle at puncture wound sites. *Ddc* .47 reporter expression accumulates around punctures to high levels at 4–6 hours post-wounding. In arm>*Flo-2 embryos*, *Ddc* .47 wound reporter expression is not detected around punctures at any time post-wounding. (B) After wounding with needles loaded with trypsin, wild type (*wt*) late stage embryos dramatically activate *Ddc* .47-GFP reporter expression throughout most or all epidermal cells at 6 hours post-wounding. In late stage embryos overexpressing *Flo-2 (arm>Flo-2)*, the trypsin-induced *Ddc* .47 wound reporter activation is completely repressed. (C,D) Embryos with the *Ddc* .47 epidermal wound reporter that overexpress *Flo-2* in stripes using the *en*-GAL4 driver (*en>Flo-2*) show inhibition of both wound-induced (C) and trypsin-induced (D) activation of the *Ddc* .47 wound reporter in all epidermal cells, not just the epidermal cells that overexpress *Flo-2* (see Figure S2B). Arrows show wound sites. Dashed lines in the data panels mark the outlines of embryos. Scale bar = 50 µM.

With *Flo-2* loss of expression resulting in widespread activation of epidermal wound genes, we wished to test whether overexpression of *Flo-2* would have an effect on wound gene activation. This was accomplished using a fly line in which the *Flo-2* cDNA was fused to a UAS promoter, in combination with an *arm*-GAL4 driver [44]. *armadillo* (*arm*), the *Drosophila* homolog of ß-catenin, is expressed ubiquitously in embryos, and the *arm*-GAL4 driver can induce high levels of UAS-*Flo-2* expression beginning at stage 10 of embryonic development (Figure S2A, [45]). Ubiquitous high levels of *Flo-2* inhibit the activation of the *Ddc* .47 and *ple*-WE1 epidermal wound reporters around wound sites (Figure 4A, Figure S3A). To test whether the inhibition caused by overexpression of *Flo-2* was cell autonomous, we used *en*-GAL4 to drive high levels of *Flo-2* in the *engrailed* (*en*), posterior compartment of each embryonic segment (Figure S2B, [46]). Overexpression of *Flo-2* using *en*-GAL4 is sufficient to silence the activation of the *Ddc* .47 and *ple*-WE1 epidermal wound reporters in all cells near a wound, even in those that do not produce *Flo-2* at higher levels (Figure 4C, data not shown). The lack of any activation of the epidermal wound reporter in the *en>Flo-2* overexpression experiments suggests that *Flo-2* can act cell non-autonomously, at least at short range, to inhibit the ability of cells to respond to wound signals. Higher levels of *Flo-2* do not appear to be toxic, as overexpression of *Flo-2* with either the *arm*-GAL4 or the *en*-GAL4 drivers does not obviously alter embryonic development, and animals so treated survive to produce viable and fertile adults (data not shown).

Although we do not know the signaling mechanisms that allow cells 5–10 cell diameters from a wound site to sense the presence of an epidermal break and activate wound gene transcription, one system that might be involved is an activation of serine protease cascades. Serine proteases regulate the production of some localized developmental signals [47], infectious innate immune signals [9], and activate localized melanization around wounds [48]. We tested whether a serine protease, trypsin, would be sufficient to induce widespread activation of epidermal wound reporter genes when injected into late stage embryos. Injection of trypsin into the body cavity (or into the perivitelline space) of stage 16 embryos results in a global activation of *Ddc* .47 and *ple*-WE1 epidermal wound reporters (Figure 4B, Figure S3B). This trypsin treatment does not appear to result in widespread epidermal cell death, nor is the epidermal paracellular barrier—which prevents diffusion of all but very small molecules through epithelia—breached when trypsin is injected into the perivitelline space of stage 16 embryos (R.P., unpublished results). Although trypsin is sufficient to activate the *ple* and *Ddc* wound reporter genes, as yet we have no current evidence that a specific endogenous serine protease is required to activate epidermal wound-induced transcriptional responses. Strikingly, overexpression of *Flo-2* under the control of either *arm*-GAL4 or *en*-GAL4 is sufficient to inhibit trypsin-induced activation of *Ddc* .47 or *ple*-WE1 wound reporters throughout the entire embryonic epidermis (Figure 4B, 4D; Figure S3C, S3D). This finding suggests that puncture-induced and trypsin-induced activation of wound genes might act through a common pathway that can be inhibited by overexpression of *Flo-2*. The inhibition of protease-induced wound reporter gene activation observed with *en>Flo-2* overexpression is consistent with the idea that *Flo-2* can act cell non-autonomously.

**Figure 5:**
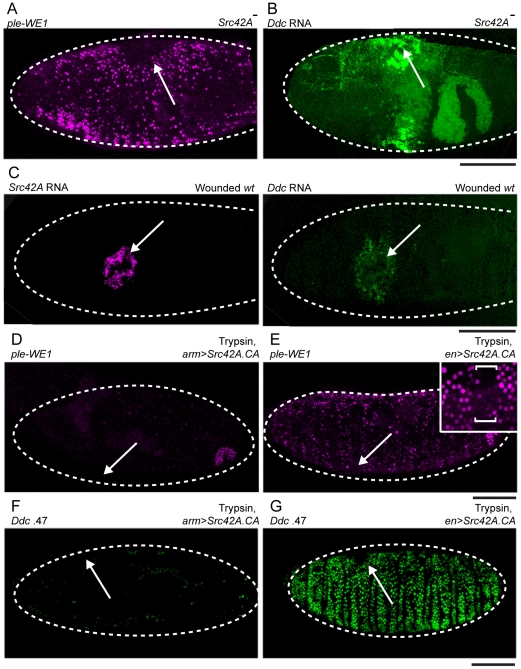
*Src42A* functions to inhibit widespread epidermal wound reporter activity after wounding. (A) Fluorescent confocal image of *ple*-WE1-dsRed epidermal wound reporter activity in a late stage *Src42A* mutant embryo. At 6 hours after wounding, the *Src42A* mutants show activation of the *ple*-WE1 wound reporter in a very broad area of the embryonic epidermis surrounding the wound site (compare with Figure 1B). (B,C) Confocal images of fluorescent *in situ* hybridization experiments. (B) 30-minutes after wounding, *Src42A* mutant embryos show accumulation of *Ddc* transcripts in a widespread zone of epidermal cells around the wound site (compare with *Ddc* expression in wounded wild type (*wt*) in panel C- right side). (C) Using double *in situ* hybridization with probes labeled with different fluorophores [9] (both images in C are taken from the same wild type (*wt*) embryo 30 minutes after wounding), we observed that *Src42A* transcripts are activated to high levels around wound sites (left panel), and in a slightly smaller zone than *Ddc* transcripts at the same stage. (D,E) Fluorescent confocal images of *ple*-WE1-dsRed epidermal wound reporter activity in late stage embryos (6 hours post-wounding) after body cavity injection of trypsin. (D) Ubiquitous expression with *Src42A.CA* inhibits the trypsin-induced *ple*-WE1 reporter in late stage embryos (compare with Figure S3B). (E) Overexpression of *Src42A.CA* in stripes with the *en*-GAL4 driver only inhibits *ple*-WE1 reporter expression in the cells where *en*-GAL4 is activating high levels of *Src42A.CA* protein expression. The insert provides higher magnification, and the bracket shows the stripe of cells where *Src42A.CA* is overexpressed by the *en*-GAL4 driver. (F,G) Confocal images of *Ddc* .47 epidermal wound reporter activity in late stage embryos (6 hours post-wounding) after body cavity injection of trypsin. (F) Ubiquitous expression with *Src42A.CA* inhibits the trypsin-induced *Ddc* .47 wound reporter in late stage embryos. (G) Overexpression of *Src42A.CA* in stripes with the *en*-GAL4 driver only inhibits *Ddc* .47 reporter expression in the cellular stripes where *en*-GAL4 is activating high levels of *Src42A.CA* protein expression. Arrows mark sites of wounds. Dashed lines in the data panels mark the outlines of embryos. Scale bar = 50 µM.

Similar to *Flo-2* mutants, mutants in *Drosophila Src42A* show more widespread activation of the *ple* wound reporter or *Ddc* transcription in epidermal cells after localized punctures (Figure 5A, 5B). Since previous research has uncovered functional and biochemical interactions between Flotillins and Src family tyrosine kinases [33], [49], we decided next to focus on the relationships between Flo-2 and Src42A in the regulation of epidermal wound response genes. Src42A is the *Drosophila* homolog of vertebrate c-Src [50]. Src family kinases were found to play important roles in several signaling pathways [51]. Like *Flo-2*, *Src42A* is itself a wound response gene; *Scr42A* transcripts accumulate to high levels in cells surrounding wound sites in wild type embryos (Figure 5C). *Src42A* transcription is also globally activated in all epidermal cells in wounded *Flo-2* mutant embryos, although the converse is not true, as *Flo-2* transcript levels are unchanged in *Scr42A* mutants (data not shown). In this sense at least, *Flo-2* wound-dependent transcriptional activation is not behaving as other wound-induced genes like *Ddc* and *ple*, which show widespread wound-induced transcription in *Src42A* mutants.

Similar to *Flo-2*, overexpression of *Src42A.CA* (a constitutively activated form, [52]) with *arm*-GAL4 inhibits both the local puncture, as well as trypsin-induced, activation of the *Ddc* .47 and *ple*-WE1 epidermal wound reporters (Figure 5D, 5F; data not shown). In contrast to *Flo-2*, overexpression of *Src42A.CA* in stripes using *en*-GAL4 inhibits the trypsin-induced activation of the *ple*-WE1 and *Ddc* .47 epidermal wound reporters only in the cells where *en*>*Src42A.CA* is over-expressed (Figure 5E, 5G). Thus, overexpressed *Src42A.CA* acts cell autonomously to inhibit epidermal wound reporter activity. Embryos with deletion mutations that eliminate other Src family tyrosine kinases, e.g. *Btk29A* (Tec homolog), *shark* (Syk homolog), *hopscotch* (JAK homolog), and *minibrain* (DYRK homolog) all had normal, localized *ple*-WE1 wound reporter activity (Table 1). This suggests that the *Src42A* inhibition of epidermal wound reporter activity is specific, and not a general property of Src family tyrosine kinases.

To test whether chemical inhibition of Src function would result in widespread wound reporter activation, we simultaneously wounded and injected one such chemical inhibitor, SU6656 [53], into the body cavity. This treatment induced widespread, patchy expression of the *Ddc* .47 and *ple*-WE1 wound reporters throughout the embryonic epidermis (Figure S4A, S4B). This widespread activation of wound reporter genes after inhibition of *Src* kinase function was not suppressed by overexpression of *Flo-2* using the *arm*-GAL4 driver (Figure S4A, S4B).

**Figure 6:**
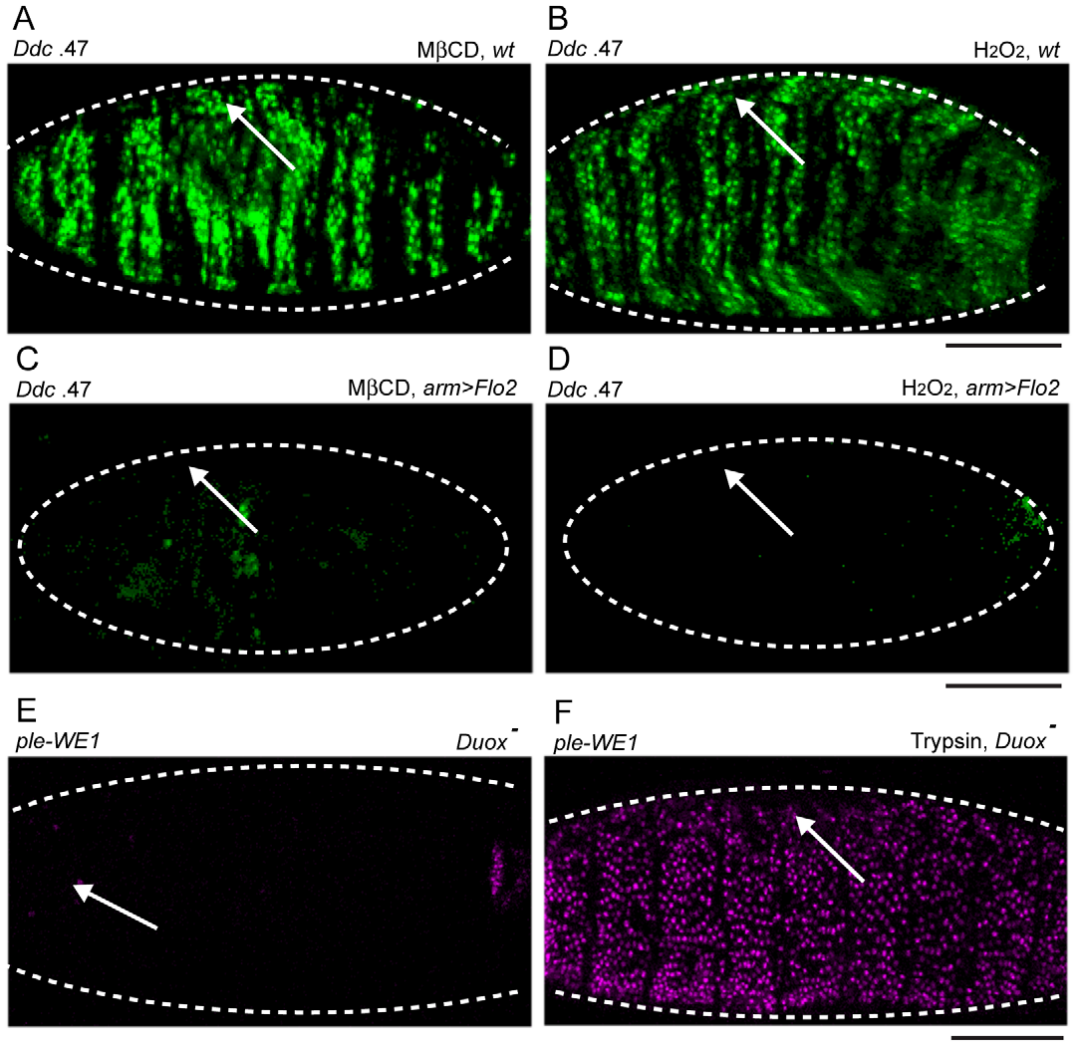
Chemical activation of the epidermal wound reporters by H_2_O_2_ and methyl-ß-cyclodextrin (MßCD) is consistent with the affects of *Duox* and *Flo-2* mutations on wound reporter expression. (A–F) Fluorescent confocal images of *Ddc* .47 or *ple*-WE1 epidermal wound reporters (all embryos shown are 6 hours post-wounding). Body cavity injection of MßCD (A) and H_2_O_2_ (B) into wild type (*wt*) embryos activates the *Ddc* .47 wound reporter throughout the entire epidermis. Overexpression of *Flo-2* with an arm-GAL4 driver (*arm>Flo-2*) can inhibit both MßCD (C) and H_2_O_2_-induced (D) activation of the *Ddc* .47 wound reporter (or the *ple*-WE1 wound reporter, not shown). (E) In *Duox* mutant embryos, wound-induced *ple*-WE1 reporter expression is not detected (F) However, in *Duox* mutant embryos treated with trypsin *ple*-WE1 reporter expression is detected in all epidermal cells. Scale bar = 50 µM.

Flo-2 is associated with, and may stabilize the Flotillin-dependent fraction of lipid rafts in membranes [33]. Therefore, we were prompted to test whether chemicals that disrupt lipid rafts might influence the spread of wound reporter activation in the epidermis. One chemical that inhibits lipid raft formation is methyl-ß-cyclodextrin (MßCD), which depletes cholesterol (and other similar lipids) from cell membranes, and can influence many intracellular signaling pathways [54], [55], [56]. Simultaneous wounding and injection of MßCD into the body cavity of *Drosophila* embryos is sufficient to activate the *Ddc* .47 and *ple*-WE1 wound reporters throughout the entire embryonic epidermis (Figure 6A and data not shown). This result suggests that the effect of reducing functional *Flo-2* on the wound response can be mimicked by more severe disruptions in lipid raft organization and membrane composition.

One wound-induced signal that can function to attract blood cells to the site of clean wounds in zebrafish and *Drosophila* embryos is Hydrogen Peroxide (H_2_O_2_) [57], [58], [59]. We wished to test whether injection of H_2_O_2_ into the body cavity of *Drosophila* embryos was sufficient to activate the *Ddc* .47 and *ple*-WE1 epidermal wound reporters in embryos, and found that a wide range of concentrations of H_2_O_2_ could activate the *Ddc* .47 and *ple*-WE1 wound reporters in most or all epidermal cells (Figure 6B, and data not shown). Interestingly, *Flo-2* or *Src42A.CA* overexpression is sufficient to inhibit the both MßCD and H_2_O_2_ activation of the *Ddc* .47 and *ple*-WE1 epidermal wound reporters (Figure 6C, 6D; Figure S4C, S4D, and data not shown). These results suggest that high levels of *Flo-2* or *Src42A.CA* are potent inhibitors of chemically-induced transcriptional activation of these epidermal wound reporters. To determine whether H_2_O_2_ production was required for the induction of epidermal wound reporters, we tested a *Drosophila* mutant in the gene for the Dual oxidase protein (Duox), the enzyme responsible for the production of H_2_O_2_, [57]. We found that *Duox* mutant embryos show a dramatically decreased activation of the *ple*-WE1 epidermal wound reporter (Figure 6E). However, in the *Duox* mutant background, trypsin injection is still sufficient to activate the *ple*-WE1 reporter throughout the epidermis (Figure 6F), suggesting H_2_O_2_-induced wound signaling might be upstream of, or in parallel to, a serine protease-dependent activation of epidermal wound reporters.

## Discussion

*Drosophila melanogaster* wound healing is an example of a regenerative process, which requires localized epidermal cytoskeletal changes, and localized wound-induced changes in epidermal transcriptional activity [60]. Our genetic screen with wound-dependent reporters has allowed us to identify novel components that regulate the localized transcriptional response to wounding in epidermal cells. This research identifies seven genes that are required to either activate (*Duox* and *ghost/stenosis*) or localize (*Flo-2*, *Src42A*, *wurst*, *varicose*, and *Drosophila* homolog of yeast *Mak3*) the expression patterns of epidermal wound reporters. The number of new functions involved in the delimitation of epidermal wound response near wound sites was unexpected, but indicates that considerable genetic effort is devoted to localizing the activity of transcriptional wound responses during regeneration.

One of the genes that limits the spread of epidermal wound reporters after clean epidermal punctures is *Flo-2*, as mutants of this gene show a broad expansion of epidermal wound gene activation. *Drosophila Flo-2* is itself transcriptionally activated around epidermal wound sites, consistent with an evolutionarily conserved role in regeneration after wounding. In vertebrates, *reggie-1/Flo-2* gene expression is activated in wounded fish optic neurons [30], and *reggie-1/Flo-2 and reggie-2/Flo-1* morpholino knockdowns in wounded zebrafish retinal explants reduced axon outgrowth compared to controls [61]. *Flo-2* transcriptional activation around *Drosophila* epidermal wound sites is dependent on the *grh* genetic function, which is required to activate at least a few other epidermal wound response genes [5], [25], [28]. *Flo-2* thus appears to act in the same pathway as *grh*, although it may act both downstream and upstream of *grh*, since overexpression of *Flo-2* can inhibit the activation of other *grh*-dependent wound response genes. In this respect, *Flo-2* resembles the *stit* receptor tyrosine kinase gene, which is both transcriptionally activated by Grh, as well as required for *grh*-dependent activation of other downstream wound genes [28]. Amazingly, overexpression of *Flo-2* can even inhibit the global activation of the *Ddc* .47 and *ple*-WE1 wound reporters that are induced by the serine protease trypsin, or by hydrogen peroxide. The inhibitory function of overexpression of *Flo-2* on wound induced transcription is cell non-autonomous, at least over the range of a few cell diameters, as shown by the ability of striped overexpression of *Flo-2* to silence puncture or trypsin-induced gene activation throughout the epidermis.

The only animal where *Flo-2* null mutants have so far been characterized is *Drosophila*, where *Flo-2* has been shown to regulate the spread of Wingless (Wg) and Hedgehog (Hh) signals in the wing imaginal discs [40], [41]. In the wing discs, both the secretion rate and the diffusion rate of these two lipid-modified morphogens were increased when *Flo-2* was overexpressed, and decreased when Flo-2 and Flo-1 proteins were not expressed [41]. Despite the reduced spread of Wg and Hh morphogen proteins in *Flo-2* mutant imaginal discs, adult morphology of mutants was normal, presumably because of compensatory mechanisms that occur later in development. Whereas a reduced range of activation of *wg* and *hh* long range transcription target genes was observed in *Flo-2* mutant imaginal discs, we observe a greatly increased range of wound-induced gene activation in *Flo-2* mutant embryos. This apparent discrepancy could be explained if one invokes of a long-range wound-induced inhibitory signal that in wild type embryos diffuses faster and farther than a wound activating signal, and thereby functions to limit the wound response to nearby epidermal cells [62], and that in *Flo-2* mutants this potential inhibitory signal has reduced secretion, concentration, and/or diffusion range. This notion is consistent with the cell non-autonomous effect of overexpressed *Flo-2* on inhibiting wound- or trypsin-induced gene activation. A similar scheme of controlling signal spreading has been seen in the way that *Mmp2* acts cell non-autonomously to limit FGF signaling during *Drosophila* tracheal development and branch morphogenesis [63]. It's also possible that *Flo-2* normally is required to set a global threshold that wound-induced signals must overcome in order to activate wound transcription, for example via Flo-2-dependent endocytosis/degradation of a diffusible wound signal and its receptor (perhaps the Stit RTK [28]), and that signal strength normally surpasses the Flo-2 threshold only in the vicinity of a wound. In this model, loss of *Flo-2* would result in all epidermal cells being able to exceed the wound signal threshold, and overexpression of *Flo-2* would prevent any cells from exceeding the wound signal threshold. The cell non-autonomous effects of *Flo-2* overexpression under this model might be explained by an increase in Flo-2-dependent endocytosis/degradation that rapidly depletes an activating signal from the extracellular space.

Many previous studies have documented biochemical, molecular biological, and cell biological interactions between Src family kinases and Flotillins [33], [34], [49], [64]. In *Drosophila*, lack of *Src42A* and *Flo-2* leads to expanded spread of wound gene activation, and overexpression of *Flo-2* or activated *Src42A* can inhibit wound gene activation, which is consistent with an interaction between the two functions during the process of wound gene regulation. In cultured mammalian cells, Flo-2 can be phosphorylated by Src family kinases in an extracellular signal-dependent fashion. This phosphorylation is associated with changes in the normal intracellular trafficking of Flotillin-containing membrane microdomains and vesicles [33], [35], [49]. Since overexpressed *Flo-2* in *Drosophila* can act in a cell non-autonomous fashion to inhibit wound gene activation, and overexpressed *Src42A* acts in a cell autonomous fashion to inhibit wound gene activation, one interpretation is that *Flo-2* lies genetically upstream of *Src42A* in the epidermal wound response. This hypothesis appears to be inconsistent with the vertebrate biochemical data indicating that Src kinases phosphorylate Flotillins to activate their diverse functions. However, an observation that is consistent with *Src42A* activating Flo-2 protein function, is that even when *Flo-2* is overexpressed, addition of chemical inhibitors of Src family kinases to wounded embryos, results in widespread *Ddc* .47 or *ple*-WE1 wound reporter activation. One interpretation of this results Flo-2 protein, no matter the level of expression, is inactive in the absence of *Src42A* function. Complex feedback loops involving signaling proteins being regulated by a transcription factor, while the activity of the same transcription factors is regulated by the same signaling pathway, have been observed in the control of *Drosophila* epidermal wound gene expression and reepithelialization [22], [28], so there may be similar dynamic cross-regulatory interactions between *Flo-2* and *Src42A* in the localization of the epidermal wound response, interactions not easily captured in linear genetic pathway diagrams [65].

The inhibitory effect of *Src42A* on wound gene activation suggests that it might antagonize a signaling cascade that leads to the epidermal wound response. A good candidate for such a signaling cascade is the RTK pathway involving the Stit kinase. Stit is a RET-family RTK that is required for robust activation of the *Ddc* and *stit* wound reporter genes in wounded embryos [28]. Other evidence consistent with RTK pathway importance in wound gene activation is that phosphotyrosine accumulates persistently around wound sites [5], [28], and that ERK kinase function is required for robust activation of the *Ddc* wound reporter gene [5]. Interestingly, *Src42A* has been shown to act as an inhibitor of some *Drosophila* RTK proteins (those encoded by the *torso*, *Egfr*, and *sevenless* genes) in a few different tissues during *Drosophila* development [66]. The *Flo-2* and *Src42A* functions in epidermal wound localization after clean wounding are reminiscent of the role of *Drosophila WntD* during infectious wounding. *WntD* mutants show higher levels of some antimicrobial peptide genes after septic injury of adults [67].

Previous evidence suggested that H_2_O_2_ and *Duox* could provide wound-induced inflammatory signals and antimicrobial activities [58], [59], [68], [69], [70], [71]. Our studies show that *Duox* is required to activate wound reporter genes after epidermal wounding, and that injected exogenous H_2_O_2_ is sufficient to activate widespread epidermal wound gene expression. Overexpression of either *Flo-2* or *Src42A.CA* can inhibit the H_2_O_2_ -dependent wound reporter expression, suggesting that all of these components are in a common pathway controlling the activation of epidermal wound reporters. However, the ability of trypsin injection to activate the *Ddc* .47 and *ple*-WE1 wound reporters in *Duox* mutants suggests that a serine protease might act downstream of, or in parallel to, H_2_O_2_-dependent wound signals. A recent report showed that in cultured mammalian cells, a Src kinase phosphorylates and inhibits a Flo-2-associated enzyme, peroxiredoxin-1, which results in increased stability of H_2_O_2_ [72]. This is consistent with our results placing Flo-2, Src42A, and H_2_O_2_ in a common wound signaling pathway.

Like H_2_O_2_, the injection of methyl-ß-cyclodextrin (MßCD) into wounded embryos triggers a global wound response in the epidermis. MßCD strongly depletes cholesterol and other sterols from membranes and disrupt lipid rafts [73], [74], but was also shown to remove sphingolipid-associated proteins such as Src-Family Kinases [54]. The effects of MßCD, in combination with the effects of loss of Flo-2, suggests that the integrity of lipid rafts and associated proteins are required to inhibit epidermal wound signals. In cultured cells, MßCD treatments trigger a release of EGF receptors from membrane microdomains, which increases EGFR, and perhaps other RTK, signaling in a ligand-independent manner [75]. Interestingly, in cultured keratinocytes, MßCD treatment can induce the expression of *involucrin* [76], which encodes a protein, analogous to *Drosophila* Ple/tyrosine hydroxylase, which is required for the formation of an epidermal barrier. Similarly, MßCD injections into *Drosophila* embryos might also cause an increase the levels of a wound signal produced or released from cells adjacent to the wound site, allowing more widespread transcriptional activation of wound reporter genes. Our observations that overexpression of *Src42A* or *Flo-2* can inhibit the MßCD -triggered activation of epidermal wound reporter genes suggest that high levels of these proteins might overcome lipid raft-inhibitory effects on wound signaling pathways.

Other genes (*wurst* and *varicose*) identified in the screen have phenotypes similar to *Flo-2* and *Src42A* mutants (Figure 1). *wurst* encodes an evolutionarily conserved trans-membrane protein, containing a heat shock cognate protein 70 binding domain and a clathrin binding motif [77]. *wurst* is ubiquitously expressed in embryonic epithelial cells, strongly up-regulated during endocytosis-dependent luminal clearance, and mislocalized in mutants with endocytosis defects [77]. *wurst* mutant embryos have tortuous tracheal tubes, due to a failure to properly endocytose matrix material from the tracheal lumen [77]. *varicose* encodes an evolutionarily-conserved septate junction scaffolding protein, in the Membrane Associated GUanylate Kinase (MAGUK) family [78], [79], [80]. *varicose* is expressed in epidermally-derived cells (including the hindgut and trachea) and co-localizes with the septate junction proteins, Coracle and Neurexin4 [80]. *varicose* mutant embryos develop permeable tracheal tubes and paracellular barrier defects in epithelia [79], [80]. Like *wurst* mutants, *varicose* mutants also have abnormal matrix composition in the tracheal lumen, and may also have abnormal extracellular matrix composition produced by other epidermal cells.

Another gene (*ghost*, also known as *stenosis*) identified in this screen is required for wound reporter activation like *Duox* or *grh* (Figure 1). *ghost* encodes the *Drosophila* Sec24CD homolog, a coat protein of COPII vesicles in the ER/Golgi trafficking pathway [81], [82]. Transport of cargo from the ER to the Golgi via COPII vesicles is required to achieve normal amounts of secretion of extracellular matrix proteins into the developing *Drosophila* tracheae and normal apical-basal localization of membrane proteins [81], [82], [83]. Presumably, similar secretion and membrane localization defects occur in non-tracheal epidermal cells, which account for the severe cuticle deposition defects in *ghost* (Sec24CD) mutants. It is fascinating to note that our finding that *ghost* (Sec24CD) is required for transcriptional activation of epidermal wound reporter genes is consistent with the finding that RNAi knockdowns of *Sec24C* in a planaria (*Schmidtea mediterranea*) interfered with normal regeneration after amputation wounds [84]. It is possible that the *ghost* mutants do not secrete enough wound signals, or the protein matrix necessary for the propagation of a wound signal.

Another gene required for the activation of wound reporters is *shroud* (*sro*). Based on a previous paper by Giesen et al. (2003) [85], we believed *sro* to be an allele in the *Drosophila Fos-D* isoform [25], and hypothesized that one of the *Drosophila kayak*/Fos transcription factors was required for the activation of some epidermal wound gene reporters [25]. However, as Niwa et al. (2010) [86] recently discovered, *sro*[*1*] and other *sro* point mutant alleles do not map in the *kayak*/Fos gene, but in an immediately adjacent transcription unit (*Nm-g/sro*) that encodes an enzyme in the sterol metabolic pathway that is necessary for production of ecdysone hormone. At first glance, the requirement of *sro* to activate some wound reporters suggested that these reporters rely on ecdysone signaling. This is possible, although we have tested deletions that eliminate zygotic functions of the *ecdysone receptor* gene, as well as of the *phantom* gene (which encodes another enzyme in the ecdysone synthesis pathway), and embryos that are zygotic mutants in either gene show normal activation of the *ple*-WE1 wound reporter after puncture wounding.

In summary, from our large unbiased screen, we have identified several genes that add to our understanding of the complex pathways that control the signals that activate wound response transcription near puncture wounds. At the cellular level, there appears to be a correlation between genetic functions required to localize wound-induced gene activation, and cellular functions required for endocytosis and/or apical-basal polarity. For example, one function of Flo-2 is in signal-dependent endocytosis, although Flo-2 also plays other roles in vesicular trafficking [32], [33], [34], [35], [36]. There have been many studies showing that endocytosis can regulate extracellular signaling strength and duration [87]. For example, one study found that tagged-FGF8 showed increased accumulation, spread, and target gene activation when Rab-5-mediated endocytosis was reduced in zebrafish embryos [88]. We believe that further studies on wound response signaling may provide new insights into how membrane microdomains, endocytosis of membrane receptors, and the composition and organization of the extracellular matrix, regulates the transmission of wound signals.

## Materials and Methods

### *Drosophila* Stocks

Fluorescent Balancers, Deficiencies, and Mutant alleles were obtained from the Bloomington *Drosophila* Stock Center: FKG = FM7c, P{GAL4-Kr.C}DC1, P{UAS-GFP.S65T}DC5, CKG = CyO, P{GAL4-Kr.C}DC3, P{UAS-GFP.S65T}DC7, *Flo-2{KG00210}*, *Src42A-E1*, UAS-*Src.CA*, *arm*-GAL4, *en*-GAL4, *Duox{KG07745}*, *wurst{G814}*, *varicose{03953b}*, and *ghost{KG029061}*. UAS-*Flo-2* was provided by Vladimir Katanaev. *Ddc* .47 and *ple*-WE1 were previously described [25].

### Wounding Procedure

Embryos were collected on apple juice agar plates and aged to 15–17 h at 25°C. Embryos were washed into mesh baskets, dechorionated in bleach for 1 min, then washed copiously with water. Embryos were then transferred to a clean slab of apple juice agar and aligned for 30–60 min at 18°C, transferred to slides with double-sided tape, then covered in a 1∶1 ratio of 700∶27 weight halocarbon oil. Embryos were then wounded bilaterally with fresh microinjection needles made from an automated puller mounted on a micromanipulator, allowed to recover for 3–8 h at room temperature, and visualized under fluorescent light in a compound microscope to determine wound reporter activity. At least 3 independent experiments with at least 50 successfully wounded embryos were performed. Assays involving homozygous deletion or mutant embryos were performed in parallel to heterozygous-balancer embryos. A Kr-GFP fluorescent marker on the balancer chromosome [89], was used to determine the genotype of the embryos. Assays involving UAS-GAL4 overexpression were performed in parallel to UAS-non-GAL4 controls. All embryos were impaled using a micromanipulator so that the needle protruded 1 embryo-width from the exit wound. Wound reporter responses were rated on a scale of “no activity, localized activity, or global activity.” Images were obtained by wounding embryos with microinjection needles and imaged on a Leica SP2 confocal microscope, selecting representative embryos to image. Images were resized while constraining proportions to maintain resolution. Adobe Photoshop adjustment functions were used equally on images to enhance clarity, but not to obscure, eliminate, or misrepresent any information. Original images are available on request.

### Body Cavity Injection

Individual embryos were simultaneously wounded and injected by using a syringe to expel the various solutions into the body cavity of the embryo. A Pipetman was used to load the solutions to be injected into the pulled capillary microinjection needles. Needles were broken on the side of a glass cover slip on a glass slide. Serine Protease-Trypsin from bovine pancreas was solubilized in 1 mM HCl pH 3.0 to 2 mg/mL (Sigma). Src Inhibitor-SU6656 was solubilized in 50% DMSO to 100 µM (Calbiochem). Methyl-ß-cyclodextrin (MßCD) was solubilized in 1 mM NaOH to 3 mM (Sigma). Hydrogen Peroxide-H_2_O_2_ was diluted in H_2_O to 0.6 M (Fisher). Chemical-wounded embryos were simultaneously wounded and injected with a 1∶4 ratio of 1% toluidine blue dye and solubilized compounds. Toluidine blue dye allowed for visual confirmation of solubilized compounds being injected into the body cavity. Control embryos were wounded with a broken needle containing 1∶4 ratios of 1% toluidine blue dye and solute without chemical. A wide range of chemical concentrations was tested to obtain optimal activation of the epidermal wound reporter and maintain high levels of embryo survival after body cavity injection.

### Multiplex Fluorescent *In Situ* Hybridization

Probes were generated from partial or full cDNA clones from the *Drosophila* Gene Collection [90], [91]. anti-Stitcher antibody was provided by Christos Samakovilis. Probe labeling and hybridization protocol was as described in Dave Kosman's multiplex FISH protocol [92].

## Supporting Information

Figure S1 *Flo-2* inhibits the extent of activation of multiple epidermal wound response genes. Confocal images of *in situ* hybridization and immunofluorescence experiments. All stains were done on Stage 16–17 embryos fixed 30 minutes after wounding. (A) *Flo-2* RNA stains. (B) *msn* RNA strains. (C) anti-Stitcher (Stit) antibody stains. Wild type embryos (*wt*) show enhanced expression of *Flo-2*, *msn* and Stitcher in a zone of about 3–5 cells from the edge of the wound site. *Flo-2^KG00210^* mutant embryos (*Flo-2^−^*) show no staining for *Flo-2* transcripts, but compared to wild type embryos *Flo-2* mutants have much broader domains of *msn* RNA staining and Sticher protein staining around wound sites. Arrows show wound site. Dashed lines in the data panels mark the outlines of embryos. Scale bar = 50 µM.
(TIF)

Figure S2 *Flo-2* transcript accumulation in *arm*- and *en*-GAL4 overexpression domains. Fluorescent confocal images of *in situ* hybridizations using a probe for *Flo-2* RNA. (A) In late stage embryos, *Flo-2* transcripts accumulate at much higher levels than wild type in epidermal cells when UAS-*Flo-2* expression is driven by *arm*-GAL4 (*arm>Flo-2*). (B) In late stage embryos, *Flo-2* transcripts accumulate at high levels in narrow epidermal stripes UAS-*Flo-2* expression is driven by *en*-GAL4. Dashed lines in the data panels mark the outlines of embryos. Scale bar = 50 µM.
(TIF)

Figure S3 Overexpression of *Flo-2* inhibits activation of the *ple*-WE1 epidermal wound reporter. (A) Brightfield and confocal images of *ple*-WE1 epidermal wound reporter activity in late stage embryos. At 6 hours after puncture wounding, wild type (*wt*) embryos (left panels) have a melanized clot (black arrow) and *ple*-WE1 reporter expression is enhanced around wound sites (white arrow). In late stage embryos overexpressing *Flo-2* (right panels), a similar-sized melanized clot is formed (black arrow) but enhanced *ple*-WE1 reporter expression around wound site (white arrow) is not detected. (B) Trypsin injection into the body cavity. At 6 hours after wounding, wild type (*wt*) late stage embryos activate *ple*-WE1 reporter expression throughout most epidermal cells. (C) In late stage embryos overexpressing *Flo-2* (*arm>Flo-2*) at 6 hours after wounding/trypsin treatment, the trypsin-induced *ple*-WE1 reporter activation is completely repressed. (D) Embryos overexpressing *Flo-2* in stripes with the en-GAL4 driver can inhibit wounding/trypsin-induced activation of the *ple*-WE1 epidermal wound reporter in all epidermal cells. Arrows show wound sites. Dashed lines in the data panels mark the outlines of embryos. Scale bar = 50 µM.
(TIF)

Figure S4 Src kinase effects on epidermal wound reporter activity. Fluorescent confocal images of *Ddc* .47 and *ple*-WE1 epidermal wound reporter activity in late stage embryos 6 hours after wounding+chemical injection into the body cavity. (A) Wounding+injection of a Src-Kinase inhibitor (SU6656) activates the *Ddc* .47 wound reporter in a patchy pattern throughout the epidermis of both wild type (*wt*) and *Flo-2* overexpression (*arm>Flo-2*) embryos. (B) Wounding+injection of a Src-Kinase inhibitor (SU6656) activates the *ple*-WE1 wound reporter in a patchy pattern throughout the epidermis of both wild type (*wt*) and *Flo-2* overexpression (*arm>Flo-2*) embryos. Overexpression of *Src42A* (*arm>Src42A*) can inhibit both methyl-ß-cyclodextrin (MßCD) (C) and hydrogen peroxide (H_2_O_2_) (D) activation of the *Ddc* .47 wound reporter gene expression. Arrows show wound sites. Dashed lines in the data panels mark the outlines of embryos. Scale bar = 50 µM.
(TIF)
