# Supplementary figures and images for: Duox, Flotillin-2, and Src42A Are Required to Activate or Delimit the Spread of the Transcriptional Response to Epidermal Wounds in Drosophila
Source: PLoS Genet. 2011 Dec 29;7(12):e1002424. doi: 10.1371/journal.pgen.1002424 (PMC3248467; doi:10.1371/journal.pgen.1002424)

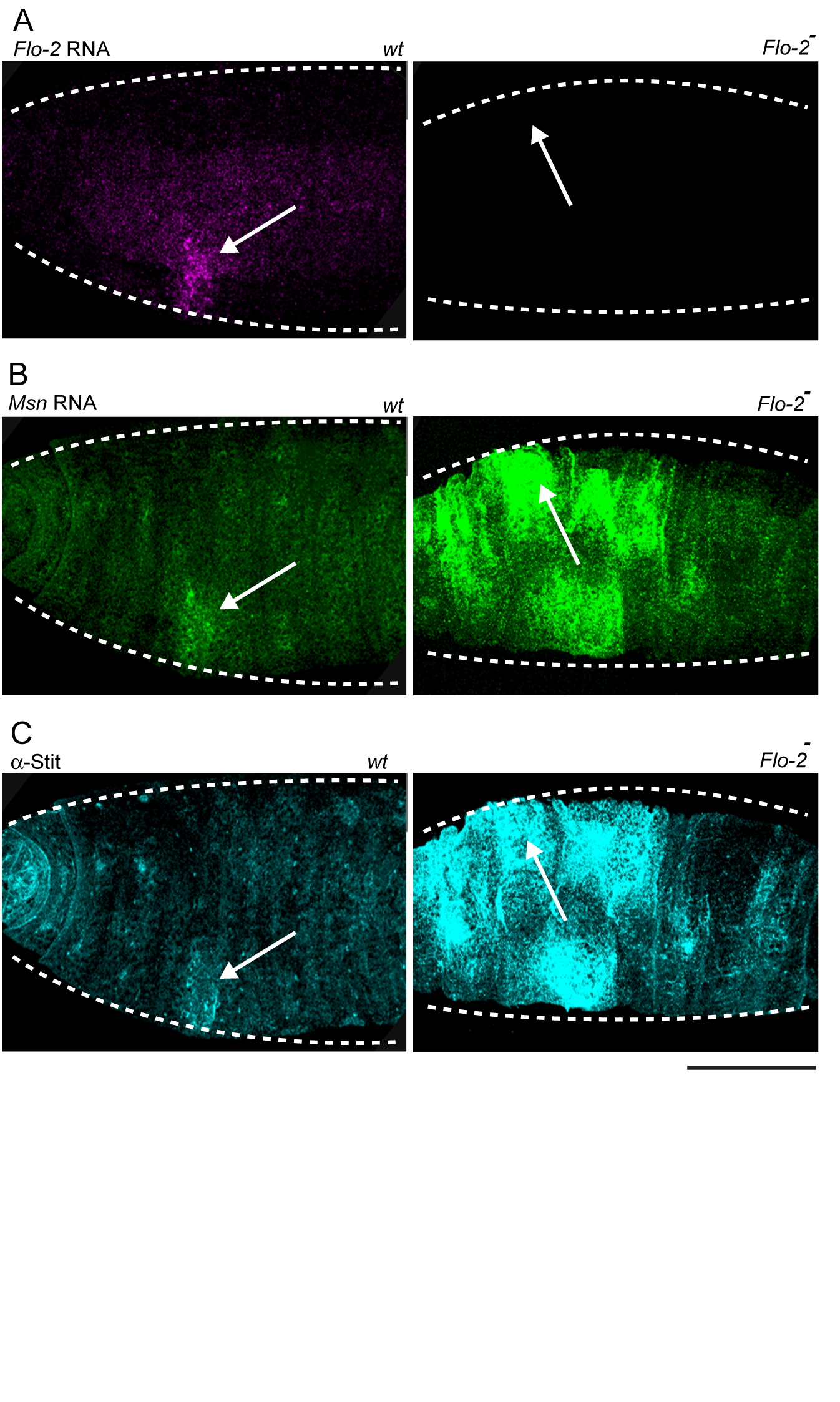

Supplement: Figure S1 — Flo-2 inhibits the extent of activation of multiple epidermal wound response genes. Confocal images of in situ hybridization and immunofluorescence experiments. All stains were done on Stage 16–17 embryos fixed 30 minutes after wounding. (A) Flo-2 RNA stains. (B) msn RNA strains. (C) anti-Stitcher (Stit) antibody stains. Wild type embryos (wt) show enhanced expression of Flo-2, msn and Stitcher in a zone of about 3–5 cells from the edge of the wound site. Flo-2KG00210 mutant embryos (Flo-2−) show no staining for Flo-2 transcripts, but compared to wild type embryos Flo-2 mutants have much broader domains of msn RNA staining and Sticher protein staining around wound sites. Arrows show wound site. Dashed lines in the data panels mark the outlines of embryos. Scale bar = 50 µM. (TIF) [file pgen.1002424.s001.tif]

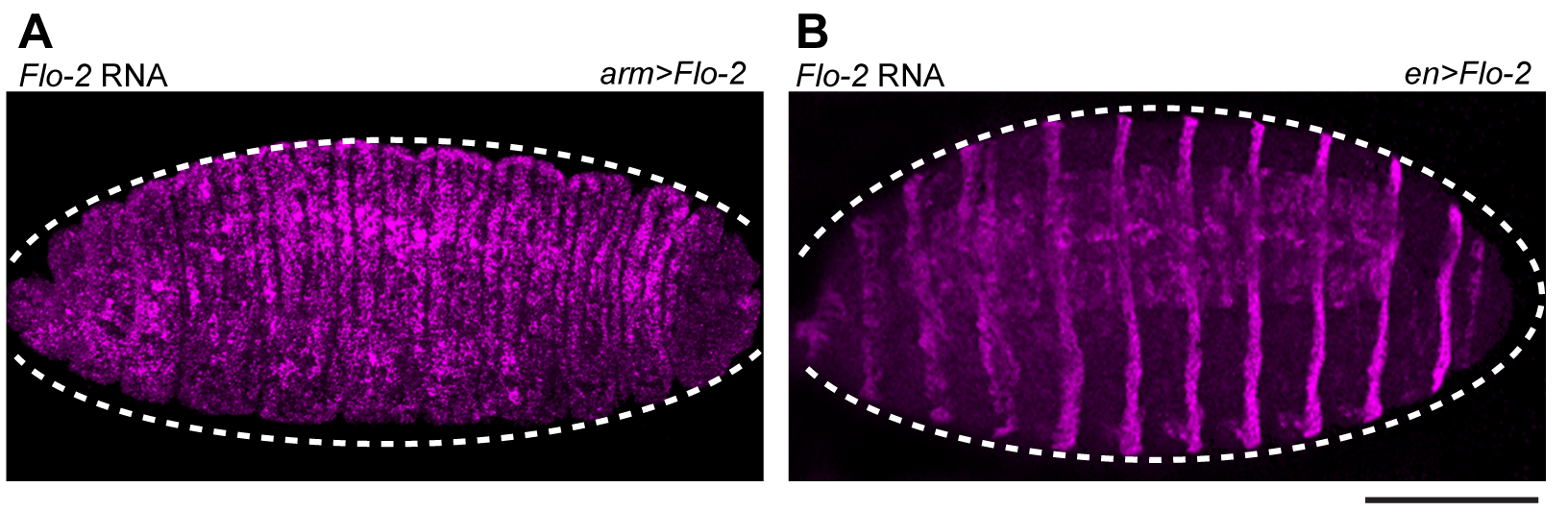

Supplement: Figure S2 — Flo-2 transcript accumulation in arm- and en-GAL4 overexpression domains. Fluorescent confocal images of in situ hybridizations using a probe for Flo-2 RNA. (A) In late stage embryos, Flo-2 transcripts accumulate at much higher levels than wild type in epidermal cells when UAS-Flo-2 expression is driven by arm-GAL4 (arm>Flo-2). (B) In late stage embryos, Flo-2 transcripts accumulate at high levels in narrow epidermal stripes UAS-Flo-2 expression is driven by en-GAL4. Dashed lines in the data panels mark the outlines of embryos. Scale bar = 50 µM. (TIF) [file pgen.1002424.s002.tif]

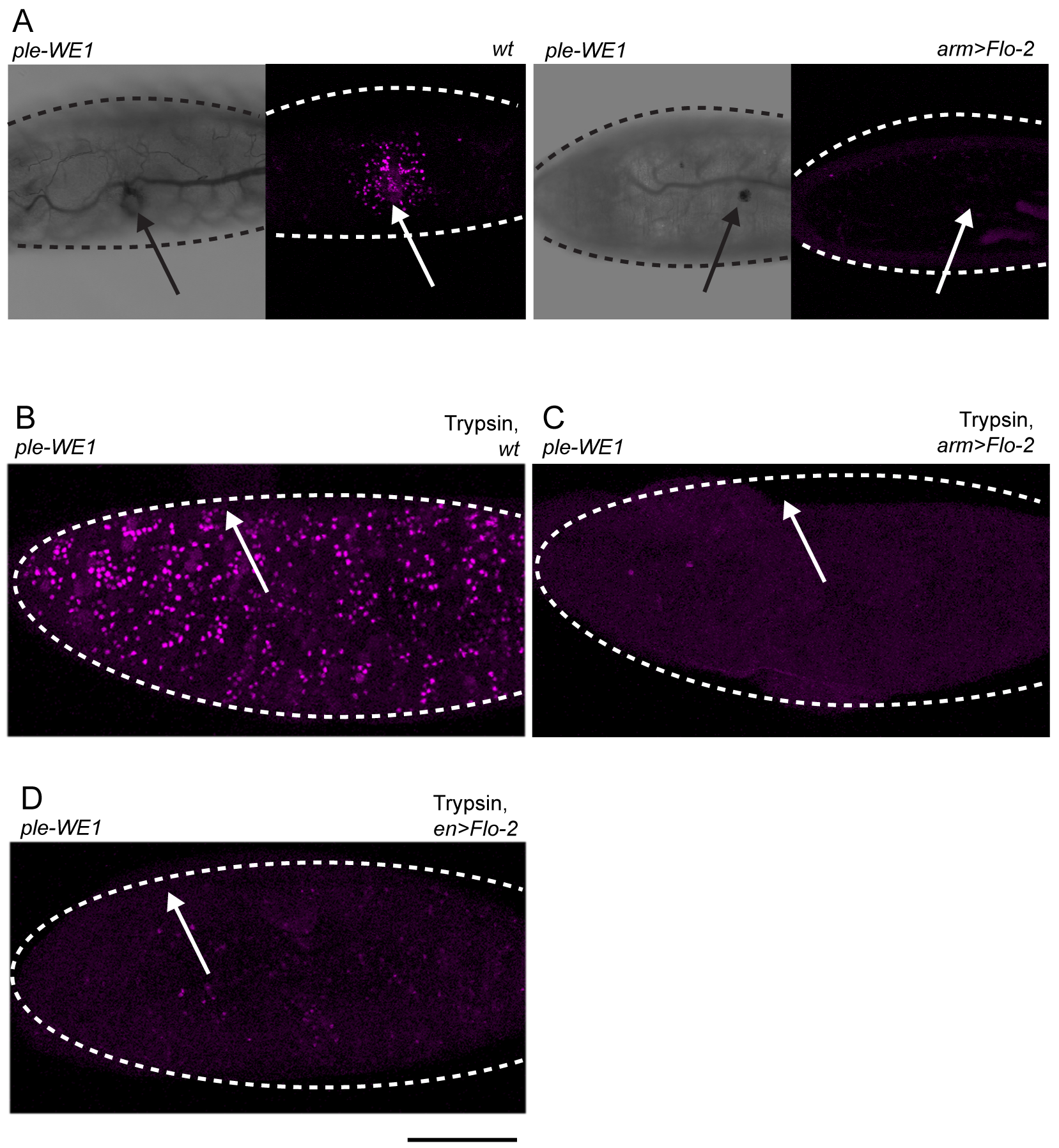

Supplement: Figure S3 — Overexpression of Flo-2 inhibits activation of the ple-WE1 epidermal wound reporter. (A) Brightfield and confocal images of ple-WE1 epidermal wound reporter activity in late stage embryos. At 6 hours after puncture wounding, wild type (wt) embryos (left panels) have a melanized clot (black arrow) and ple-WE1 reporter expression is enhanced around wound sites (white arrow). In late stage embryos overexpressing Flo-2 (right panels), a similar-sized melanized clot is formed (black arrow) but enhanced ple-WE1 reporter expression around wound site (white arrow) is not detected. (B) Trypsin injection into the body cavity. At 6 hours after wounding, wild type (wt) late stage embryos activate ple-WE1 reporter expression throughout most epidermal cells. (C) In late stage embryos overexpressing Flo-2 (arm>Flo-2) at 6 hours after wounding/trypsin treatment, the trypsin-induced ple-WE1 reporter activation is completely repressed. (D) Embryos overexpressing Flo-2 in stripes with the en-GAL4 driver can inhibit wounding/trypsin-induced activation of the ple-WE1 epidermal wound reporter in all epidermal cells. Arrows show wound sites. Dashed lines in the data panels mark the outlines of embryos. Scale bar = 50 µM. (TIF) [file pgen.1002424.s003.tif]

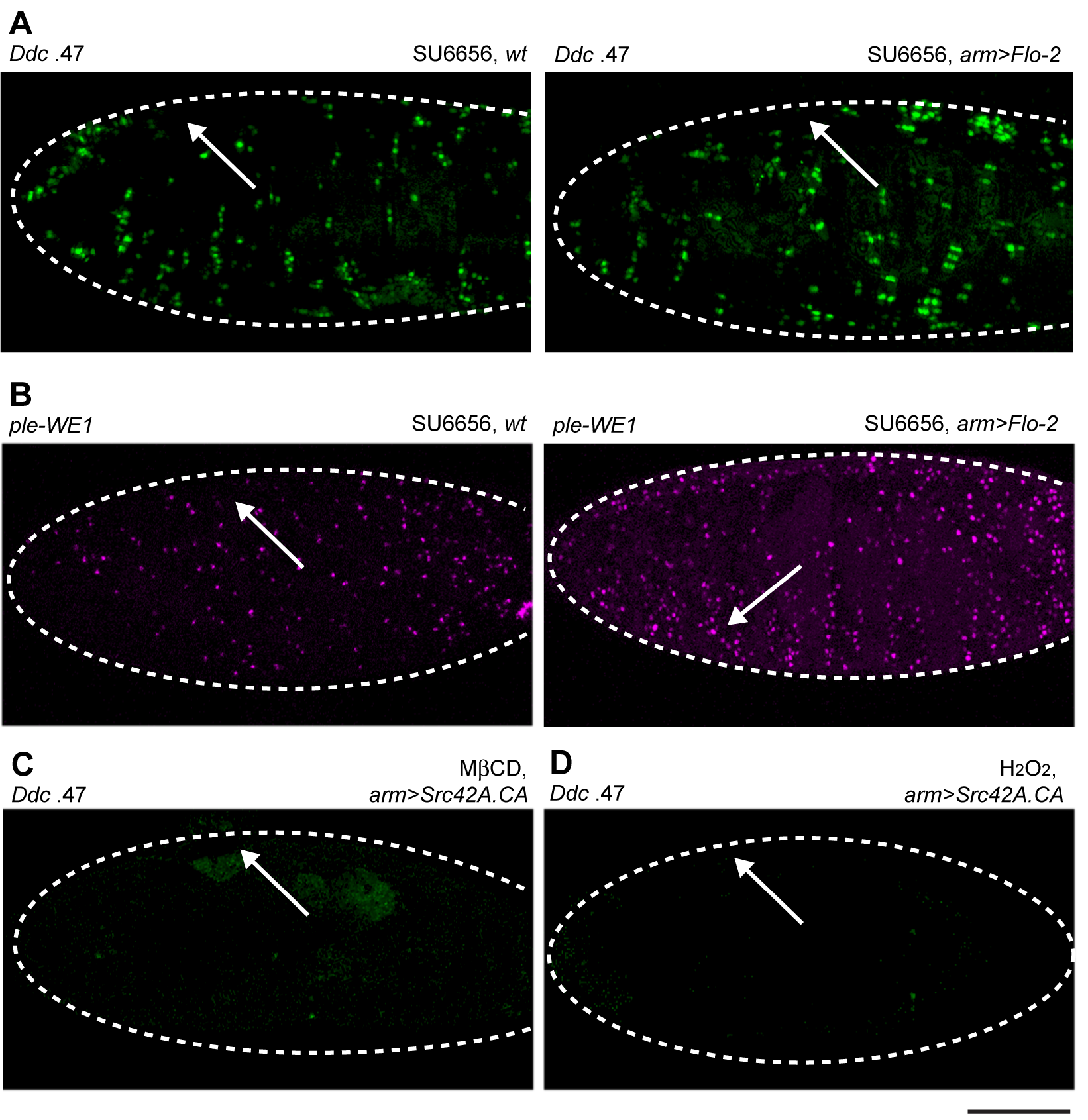

Supplement: Figure S4 — Src kinase effects on epidermal wound reporter activity. Fluorescent confocal images of Ddc .47 and ple-WE1 epidermal wound reporter activity in late stage embryos 6 hours after wounding+chemical injection into the body cavity. (A) Wounding+injection of a Src-Kinase inhibitor (SU6656) activates the Ddc .47 wound reporter in a patchy pattern throughout the epidermis of both wild type (wt) and Flo-2 overexpression (arm>Flo-2) embryos. (B) Wounding+injection of a Src-Kinase inhibitor (SU6656) activates the ple-WE1 wound reporter in a patchy pattern throughout the epidermis of both wild type (wt) and Flo-2 overexpression (arm>Flo-2) embryos. Overexpression of Src42A (arm>Src42A) can inhibit both methyl-ß-cyclodextrin (MßCD) (C) and hydrogen peroxide (H2O2) (D) activation of the Ddc .47 wound reporter gene expression. Arrows show wound sites. Dashed lines in the data panels mark the outlines of embryos. Scale bar = 50 µM. (TIF) [file pgen.1002424.s004.tif]
